# Light, questions, probes: Valentina Emiliani on combining disciplines for neurophotonics

**DOI:** 10.1117/1.NPh.10.4.040402

**Published:** 2023-05-11

**Authors:** Amanda Foust

**Affiliations:** Imperial College London, London, United Kingdom

## Abstract

Valentina Emiliani, CNRS research director at the Vision Institute in Paris, discusses her pioneering work in neurophotonics, in an interview with fellow *Neurophotonics* Editorial Board Member Amanda Foust, Senior Lecturer in Biomedical Engineering at University College London.

**Figure:**
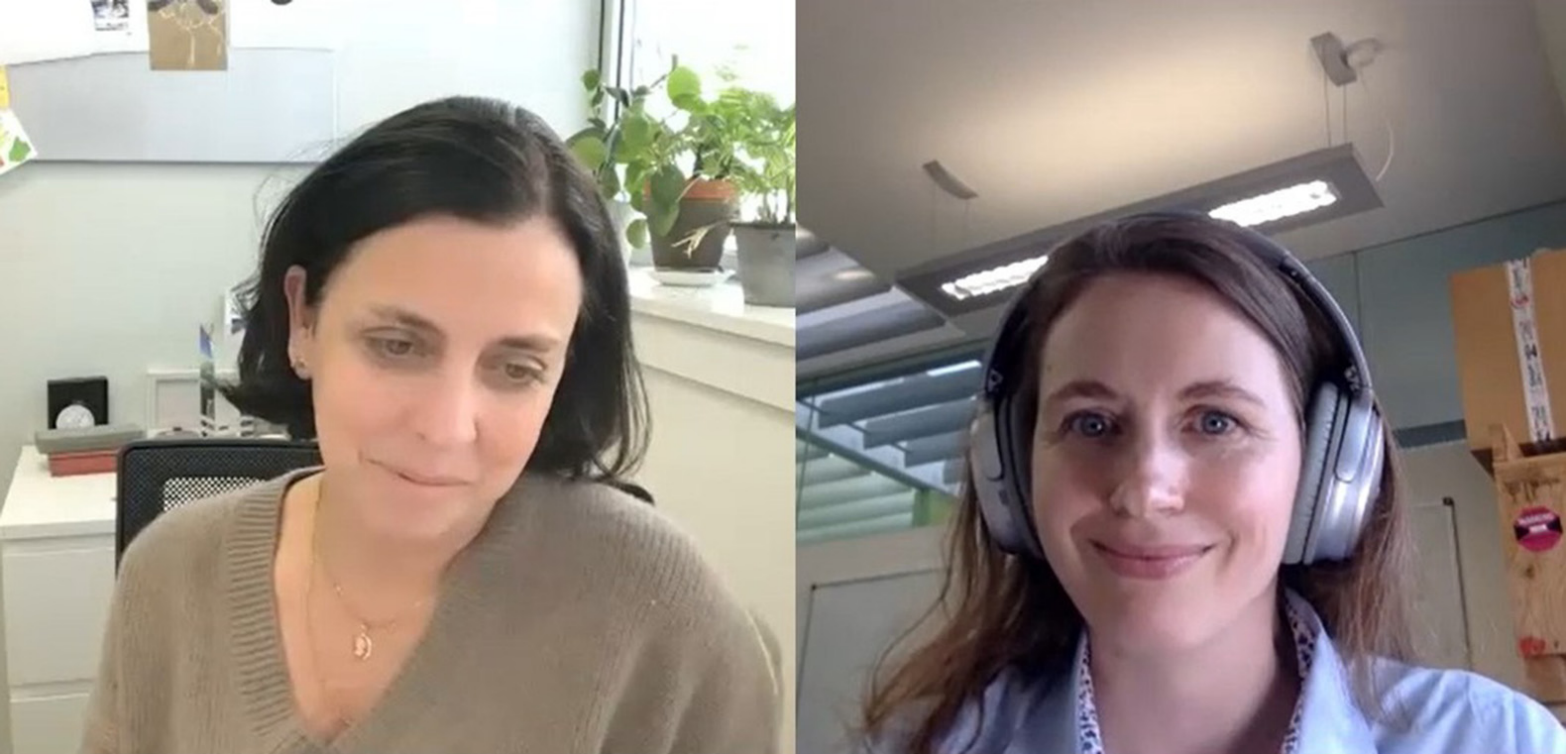
(Left) Valentina Emiliani, CNRS research director at the Vision Institute in Paris, and (right) fellow *Neurophotonics* Editorial Board Member Amanda Foust, Senior Lecturer in Biomedical Engineering at University College London. View a video recording of the interview at https://doi.org/10.1117/1.NPh.10.4.040402

